# Biomarkers of Inflammation and Radiographic Progression in Axial Spondyloarthritis: A Clinical Evaluation of Leptin, Adiponectin, TNF-α, and IL-17A

**DOI:** 10.3390/jcm14155605

**Published:** 2025-08-07

**Authors:** Alexandra-Diana Diaconu, Laurențiu Șorodoc, Cristina Pomîrleanu, Liliana Georgeta Foia, Victorița Șorodoc, Cătălina Lionte, Mara Russu, Vladia Lăpuște, Larisa Ghemiș, Codrina Ancuța

**Affiliations:** 1 Faculty of Medicine, University of Medicine and Pharmacy “Grigore T. Popa”, 700115 Iasi, Romania; 2 2nd Internal Medicine Department, “Sf. Spiridon” Clinical Emergency Hospital, 700111 Iasi, Romania; 3 2nd Rheumatology Department, Clinical Rehabilitation Hospital, 700661 Iasi, Romania; 4 Biochemistry Department, Faculty of Dental Medicine, University of Medicine and Pharmacy “Grigore T. Popa”, 700115 Iasi, Romania

**Keywords:** axial spondyloarthritis, leptin, adiponectin, TNF-alpha, IL17-A, biomarkers, disease activity, radiographic progression, syndesmophytes, personalized medicine

## Abstract

**Background/Objectives**: Axial spondyloarthritis (axSpA) is a chronic immune-mediated inflammatory disorder affecting the spine and sacroiliac joints, with variable clinical expression. This study assessed serum levels of inflammatory (TNF-α, IL-17A) and metabolic (leptin, adiponectin) biomarkers and their associations with disease activity, inflammation, structural damage, and comorbidities. **Methods**: This prospective cross-sectional study assessed 89 axSpA patients using clinical, laboratory, and radiological evaluations. Disease activity was measured using ASDAS-CRP and BASDAI scores. Radiographic damage was quantified using the Modified Stoke Ankylosing Spondylitis Spine Score (mSASSS). Serum concentrations of TNF-α, IL-17A, leptin, and adiponectin were quantified by enzyme-linked immunosorbent assay (ELISA). Clinical and imaging correlations were analyzed. **Results**: Serum leptin levels correlated significantly with higher disease activity scores, inflammatory markers (CRP, ESR), radiographic progression (syndesmophyte formation, mSASSS), and arterial hypertension. Adiponectin levels were inversely associated with disease activity, structural damage, and arterial hypertension, suggesting anti-inflammatory, bone- and cardio-protective properties. TNF-α levels showed an association with inflammatory markers and were higher in patients with peripheral enthesitis. IL-17A levels were weakly correlated with disease activity and structural severity and were significantly lower in patients with a history of anterior uveitis. **Conclusions**: Leptin and adiponectin may serve as complementary biomarkers in axSpA, reflecting both inflammatory burden and structural damage. While TNF-α and IL-17A remain key therapeutic targets, their correlation with structural changes appears limited. Biomarker profiling could support personalized disease monitoring. Longitudinal studies are needed to validate prognostic implications.

## 1. Introduction

Spondyloarthritis (SpA) represents a group of chronic inflammatory diseases with an immune-mediated origin, characterized by significant heterogeneity but sharing common features: genetic factors (HLA-B27 and non-HLA genes), clinical manifestations (musculoskeletal and extra-articular involvement), imaging findings (sacroiliitis, spondylitis, peripheral arthritis, enthesitis), and therapeutic response (favorable response to nonsteroidal anti-inflammatory drugs, NSAIDs) [1,2,3]. The term “axial spondyloarthritis (axSpA)” refers to both patients with evident structural damage in the sacroiliac joints (visible on standard radiographs), a condition known as radiographic SpA (r-axSpA) or ankylosing spondylitis (AS), as well as patients who have not yet developed structural damage, referred to as non-radiographic SpA (nr-axSpA) [4].

AxSpA is characterized by chronic inflammation affecting the sacroiliac joints and spine, leading over time to structural changes such as syndesmophyte formation and spinal ankylosis, with consequent impairment in function and quality of life [5]. Two key proinflammatory cytokines, tumor necrosis factor-alpha (TNF-α) and interleukin-17A (IL-17A), have emerged as central mediators of disease activity and targets of effective biologic therapies [6]. In addition, Janus kinase (JAK) inhibitors have recently been approved for use in axSpA, offering a new option for patients unresponsive to NSAIDs or biologics.

Despite treatment progress, there is still a lack of reliable and easily accessible biomarkers that can aid in the diagnosis, monitoring, and prognostic evaluation of axSpA. In recent years, interest has grown in inflammatory and metabolic biomarkers, including cytokines such as TNF-α and IL-17A, and adipokines like leptin and adiponectin, for their potential role in characterizing disease activity and risk of progression [7,8,9].

TNF-α plays a critical role in sustaining inflammation and promoting both bone resorption and new bone formation through complex pathways involving NF-κB, MAPKs, and Wnt signaling inhibition. Serum levels of TNF-α have been shown to correlate with clinical activity indices such as the Bath Ankylosing Spondylitis Disease Activity Index (BASDAI) and Ankylosing Spondylitis Disease Activity Score based on C-reactive protein (ASDAS-CRP), as well as with CRP and erythrocyte sedimentation rate (ESR) [10,11,12].

IL-17A, another key cytokine, promotes neutrophil recruitment and amplifies Th17 responses. Its serum concentration is also associated with inflammatory activity and response to IL-17 inhibitors [13].

Leptin and adiponectin, two major adipokines secreted by white adipose tissue, modulate both metabolism and immune responses. Leptin generally has proinflammatory effects, including activation of Th1 and Th17 cells, and has been linked in some studies to increased disease activity in axSpA, though results are inconsistent [14]. Adiponectin is widely recognized as anti-inflammatory and has shown inverse correlations with inflammatory markers and metabolic dysfunction. It modulates cytokine production and macrophage polarization and may have a protective role in bone metabolism [15,16,17].

However, data on these adipokines in axSpA remain limited and conflicting. Furthermore, many studies fail to account for important confounding factors, such as body mass index (BMI), or to explore the combined behavior of these biomarkers in relation to disease activity, imaging progression, and comorbidities.

The objective of our study was to evaluate serum levels of leptin, adiponectin, TNF-α and IL-17A in patients with axSpA and to examine their associations with disease activity, structural damage, and comorbidities. By integrating inflammatory and metabolic biomarkers into a cross-sectional cohort, we aimed to identify meaningful biomarker patterns that could support personalized risk assessment and therapeutic decision-making.

## 2. Materials and Methods

### 2.1. Study Design and Subjects

Eighty-nine consecutive patients diagnosed with axSpA (fulfilling either the ASAS 2009 criteria or the 1984 modified New York criteria) were prospectively enrolled in this cross-sectional study. All participants were assessed at the 2nd Rheumatology Department, Clinical Rehabilitation Hospital in Iasi, Romania, during 2023–2024.

### 2.2. Clinical, Laboratory, and Radiographic Assessments

Clinical, laboratory, and imaging data were systematically collected for all patients at baseline through standardized assessments.

Demographic and general health data included age, sex, BMI, smoking status (current or former), and relevant comorbidities, including arterial hypertension, dyslipidemia, and diabetes mellitus.

AxSpA-related variables comprised HLA-B27 status, family history of axSpA, disease duration, treatment, and history of peripheral arthritis or enthesitis. Extra-articular manifestations, such as anterior uveitis, psoriasis, and inflammatory bowel disease (IBD), were also reported.

Functional status was evaluated using two validated measures of spinal mobility in axSpA patients: the Schober index and the fingertip-to-floor distance.

Disease activity was measured using the ASDAS-CRP and BASDAI scores at baseline. According to the ASDAS score, disease activity was defined as follows: inactive disease (ASDAS < 1.3), low disease activity (ASDAS ≥ 1.3 and <2.1), high disease activity (ASDAS ≥ 2.1 and <3.5), and very high disease activity (ASDAS ≥ 3.5). According to the BASDAI score, low disease activity was defined as a BASDAI score < 4, while high disease activity was characterized by a BASDAI score > 4.

Pelvic radiographs (anteroposterior view) were acquired at baseline for the diagnosis and staging of axSpA. Radiographs of the lumbar and cervical spine (lateral projection) were also obtained, and previous spinal radiographs taken two years prior to inclusion in the study were reviewed.

Structural spinal damage was assessed using the modified Stoke Ankylosing Spondylitis Spine Score (mSASSS), which evaluates the anterior vertebral corners from the lower edge of C2 to the upper edge of T1 and from the lower edge of T12 to the upper edge of S1. Lesions were scored as follows: squaring, erosion, or sclerosis = 1 point; non-bridging syndesmophyte = 2 points; and bridging syndesmophyte = 3 points. Radiographic progression over the two-year interval was defined as an increase of ≥2 mSASSS points in at least one vertebral corner. Based on spinal radiographic findings, patients were stratified into three groups according to syndesmophyte severity: none, low burden, and moderate to severe burden, as determined by mSASSS criteria.

For all patients, inflammatory biomarkers, CRP and ESR, were measured using standard laboratory methods (latex-enhanced immunoturbidimetric assay for CRP; Westergren method for ESR). In addition, as part of the metabolic assessment, total cholesterol, HDL and LDL cholesterol, and triglycerides were measured in all patients.

### 2.3. Serum Biomarkers

Serum levels of metabolic (leptin, adiponectin) and inflammatory biomarkers (IL-17, TNF-α) were measured using specific enzyme-linked immunosorbent assay (ELISA) kits, following the manufacturer’s protocols (BioVendor, Heidelberg, Germany). The intra- and inter-assay coefficients of variation were 6.7% and 8.1% for leptin, 6.6% and 7.5% for adiponectin, 4.8% and 6.2% for IL-17, and 5.1% and 7.3% for TNF-α, respectively. The detection ranges were 0.17–50 ng/mL for leptin, 0.7–100 μg/mL for adiponectin, 0.5–100 pg/mL for IL-17, and 0.2–50 pg/mL for TNF-α. Samples exceeding these limits were reanalyzed after appropriate dilution. All procedures were carried out in the Biochemistry Laboratory of the “Grigore T. Popa” University of Medicine and Pharmacy, Iași, Romania, in accordance with the manufacturer’s instructions. Venous blood was collected in plain tubes without anticoagulant, allowed to clot at room temperature, and centrifuged at 3000 rpm for 10 min. The resulting serum was separated, aliquoted, and stored at −80 °C until analysis. Prior to testing, ELISA plates and reagents were brought to room temperature. For each biomarker, serum samples (either undiluted or appropriately diluted based on preliminary concentration estimates), along with calibration standards and assay-specific reagents, were added to wells pre-coated with capture antibodies. After incubation at 37 °C for a time specified in the protocol (usually 1–2 h), the plates were washed thoroughly with wash buffer to remove unbound material. A detection antibody conjugated to horseradish peroxidase was then added, followed by a second incubation and washing step. Color development was initiated by adding a tetramethylbenzidine substrate solution, and the enzymatic reaction was stopped after the prescribed time using sulfuric acid. The absorbance was measured at 450 nm using a microplate reader. All determinations were performed in duplicate, and biomarker concentrations were calculated based on standard calibration curves.

### 2.4. Statistical Analyses

Values are presented as mean ± standard deviation (SD). The analysis focused on exploring associations and correlations between biomarkers and various clinical, functional, and laboratory parameters. Depending on data distribution, the Kruskal–Wallis, Mann–Whitney U, and independent-sample *t*-tests were used for comparisons between groups. Categorical variables were analyzed using the chi-squared (χ^2^) test. Correlation analyses were performed using Pearson correlation coefficients. Correlations were interpreted as negligible (r ≤ 0.25), low (0.25 < r ≤ 0.5), moderate (0.5 < r ≤ 0.75), high (0.75 < r ≤ 0.9), and very high (r > 0.9), with values below 0.3 generally considered not meaningful. Statistical significance was set at *p* < 0.05. All analyses were conducted using SPSS version 29.0 for Windows.

## 3. Results

### 3.1. Patient Demographics, Disease-Related Characteristics, and Comorbidities

Patient demographics, disease-related characteristics, comorbidities, and serum levels of inflammatory and metabolic biomarkers are summarized in Table 1.

The study included 89 patients with axSpA, with a mean age of approximately 50 years, and a male predominance (~72%). About half were HLA-B27 positive, and the median disease duration was around 9 years. Common comorbidities included arterial hypertension (33%), dyslipidemia (36%), and diabetes mellitus (17%). Extra-articular manifestations such as uveitis, IBD, and psoriasis were also reported in a minority of cases. Inflammatory activity was moderate, with average ASDAS-CRP of 2.5 and BASDAI around 3.1. Structural spinal damage was notable, with a mean mSASSS score of 24, and approximately 44% of patients had syndesmophytes on radiography. Regarding treatment, nearly 30% received NSAIDs, 43% were on anti-TNF therapy, and smaller proportions received anti-IL-17A, JAK inhibitors, or DMARDs.

### 3.2. Biomarker Results

#### 3.2.1. Leptin in axSpA

In the overall cohort, the mean serum leptin concentration was 20.80 ± 15.52 ng/mL. Gender-stratified analysis demonstrated significantly higher levels in women (24.80 ± 14.75 ng/mL) than in men (19.23 ± 15.65 ng/mL; *p* = 0.048).

Serum leptin did not differ by HLA-B27 status (*p* = 0.723). In contrast, a weak but significant positive correlation emerged between leptin and BMI (r = 0.213, *p* = 0.045), with a stepwise rise in mean values across BMI categories (Supplementary Figure S1).

**Figure 1:**
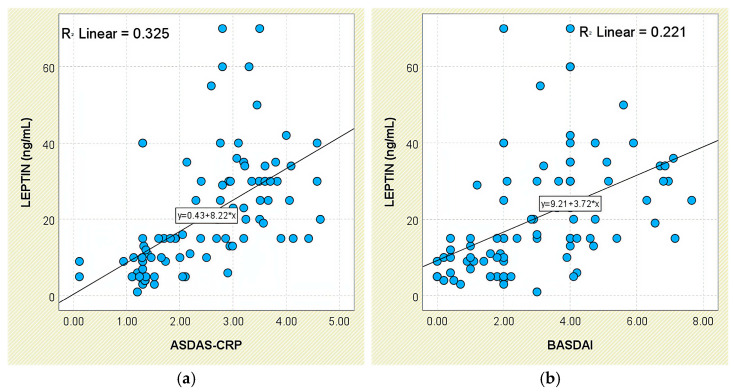
Correlation between serum leptin levels (ng/mL) and disease activity scores in patients with axSpA. (**a**) Leptin vs. ASDAS-CRP; (**b**) leptin vs. BASDAI.

Leptin correlated positively with disease-activity metrics. A modest yet significant linear association was noted between leptin and ASDAS-CRP scores (r = 0.325, *p* < 0.05; Figure 1a). Mean concentrations increased from 8.94 ± 8.66 ng/mL in the inactive group to 10.11 ± 4.55 ng/mL (low activity) and 29.16 ± 12.82 ng/mL (high/very high activity), as depicted in Supplementary Figure S2. Similarly, patients with BASDAI ≥ 4 had markedly higher leptin levels (28.95 ± 14.65 ng/mL) than those with BASDAI < 4 (14.73 ± 13.31 ng/mL; *p* < 0.001), and leptin correlated with BASDAI (r = 0.470, *p* < 0.05; Figure 1b).

**Figure 2:**
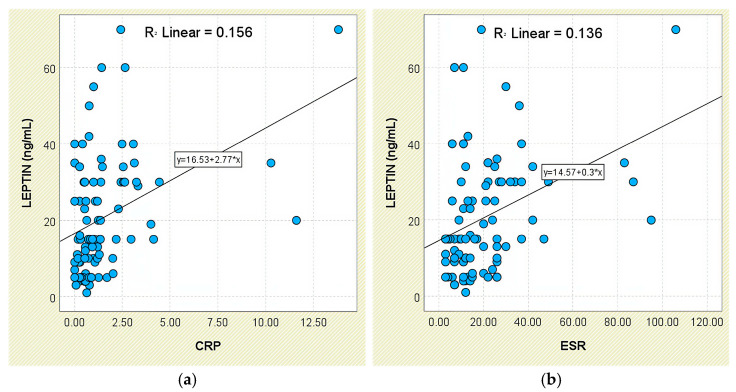
Correlation between serum leptin levels (ng/mL) and inflammatory biomarkers in patients with axSpA. (**a**) Leptin vs. CRP; (**b**) leptin vs. ESR.

Consistent findings were obtained for systemic-inflammation markers. Patients with elevated CRP exhibited higher leptin concentrations (26.23 ± 16.46 ng/mL) than those with normal CRP (16.37 ± 13.31 ng/mL; *p* < 0.001), and leptin correlated weakly with CRP (r = 0.395, *p* < 0.05; Figure 2a). A comparable pattern was seen for ESR (r = 0.369, *p* < 0.05; Figure 2b); individuals with elevated ESR had higher leptin levels (26.14 ± 14.55 ng/mL) than those with normal ESR (17.33 ± 15.27 ng/mL; *p* < 0.001).

**Figure 3:**
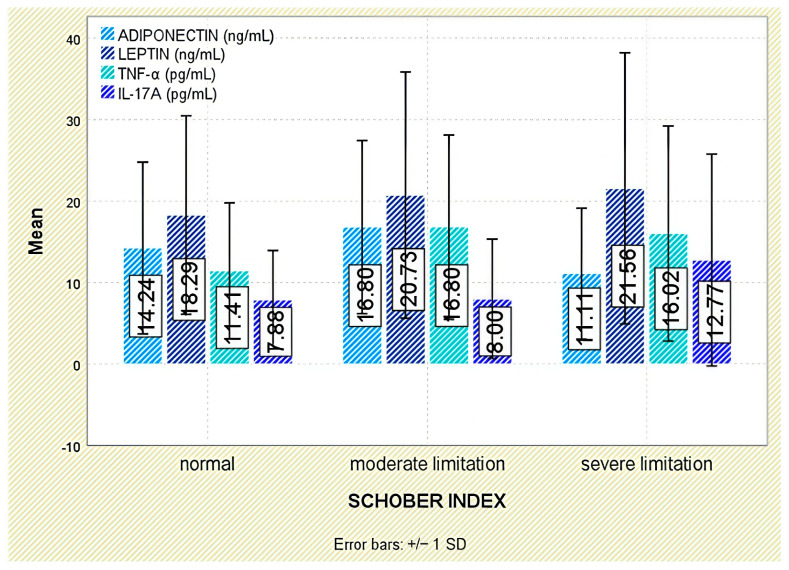
Serum levels of adiponectin, leptin, TNF-α, and IL-17A across Schober index categories. Error bars represent standard deviation.

**Figure 4:**
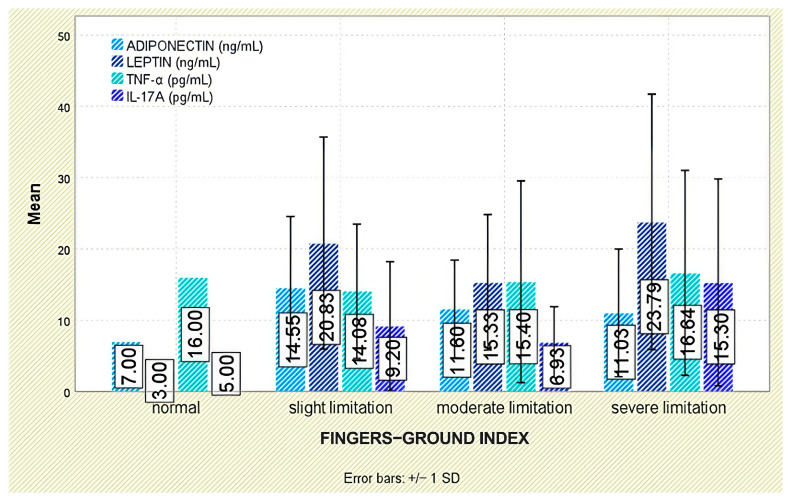
Serum levels of adiponectin, leptin, TNF-α, and IL-17A according to fingertip-to-floor test categories. Error bars represent standard deviation.

No meaningful associations emerged between leptin and functional indices (Schober test, fingertip-to-floor distance); leptin did not differ across functional-limitation strata (*p* = 0.849 and *p* = 0.294, respectively; Figure 3 and Figure 4).

**Figure 5:**
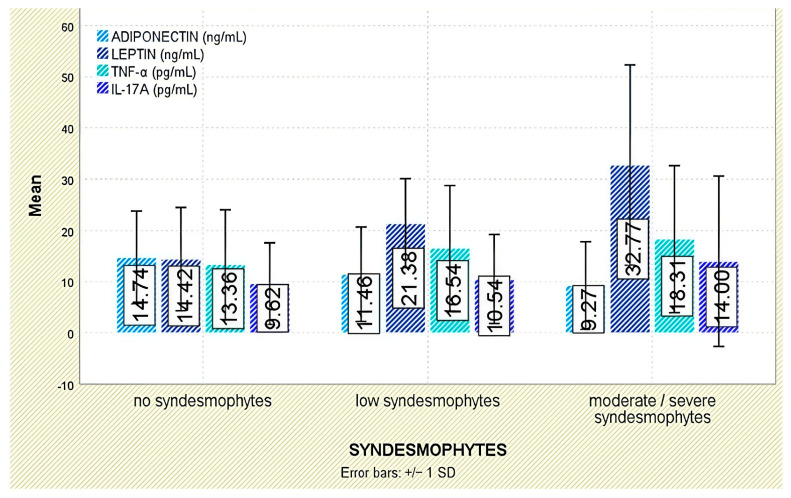
Serum levels of adiponectin, leptin, TNF-α, and IL-17A across syndesmophyte severity categories. Error bars represent standard deviation.

**Figure 6:**
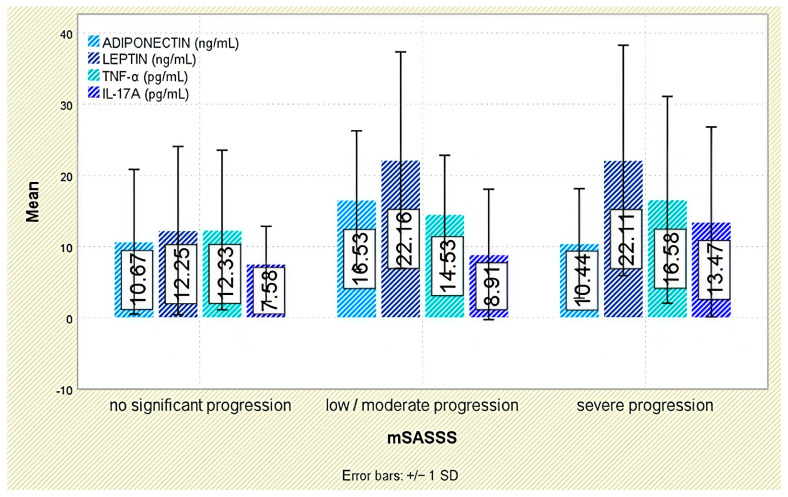
Serum levels of adiponectin, leptin, TNF-α, and IL-17A across categories of radiographic progression based on the mSASSS. Error bars represent standard deviation.

Radiographic outcomes revealed a significant positive correlation between leptin and syndesmophyte presence (r = 0.510, *p* < 0.001; Figure 5). The leptin-to-BMI ratio climbed with increasing structural damage, from a median of 0.48 (no syndesmophytes) to 1.16 (moderate–severe syndesmophytes; *p* < 0.001). Leptin was also associated with the mSASSS (*p* = 0.041; Figure 6).

**Figure 7:**
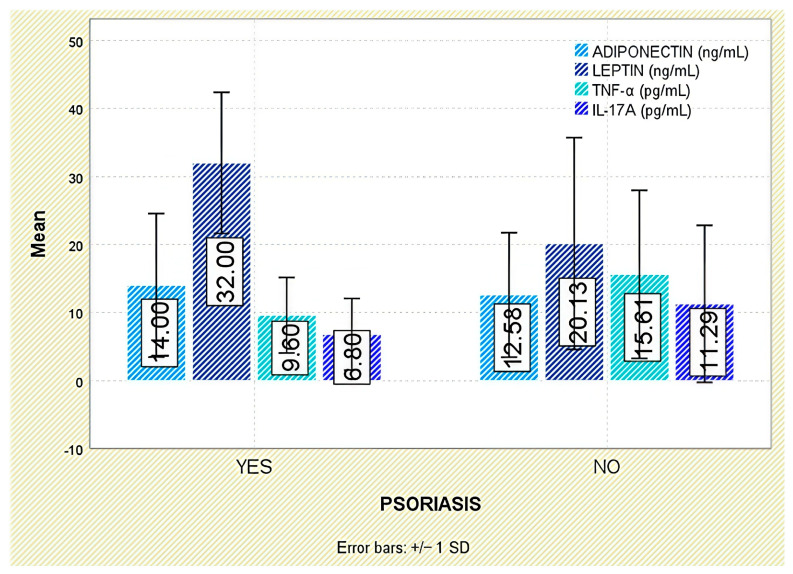
Serum levels of adiponectin, leptin, TNF-α, and IL-17A in patients with and without psoriasis. Error bars represent standard deviation.

**Figure 8:**
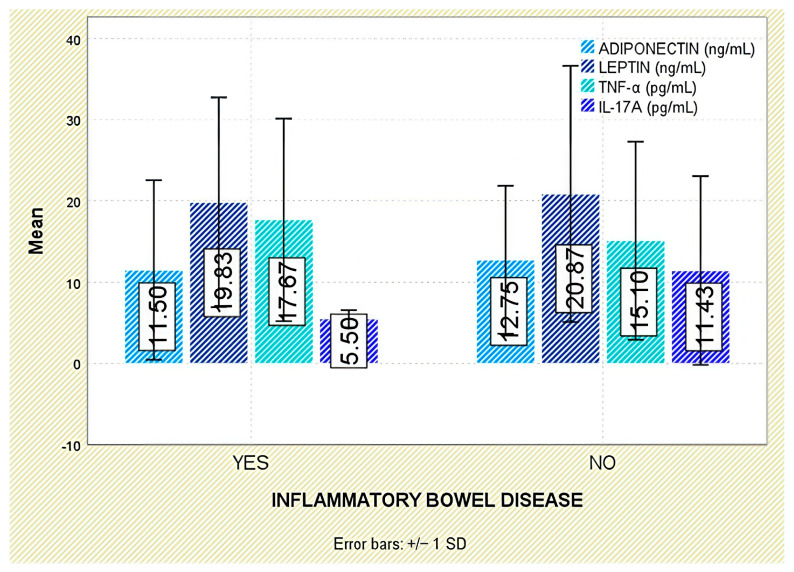
Serum levels of adiponectin, leptin, TNF-α, and IL-17A in patients with and without IBD. Error bars represent standard deviation.

**Figure 9:**
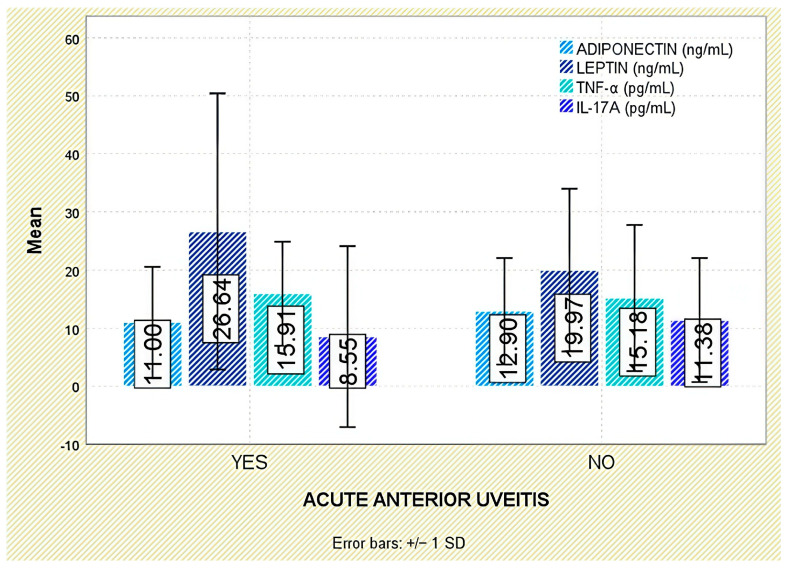
Serum levels of adiponectin, leptin, TNF-α, and IL-17A in patients with and without acute anterior uveitis. Error bars represent standard deviation.

Among extra-articular manifestations, leptin was higher in patients with psoriasis than in those without (*p* = 0.047; Figure 7); no significant differences were observed for inflammatory bowel disease or acute anterior uveitis (Figure 8 and Figure 9).

**Figure 10:**
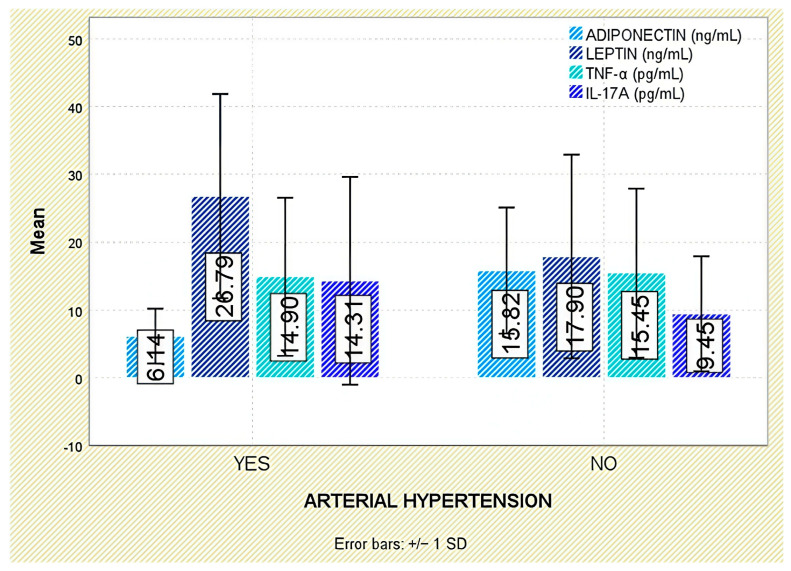
Serum levels of adiponectin, leptin, TNF-α, and IL-17A in patients with and without arterial hypertension. Error bars represent standard deviation.

**Figure 11:**
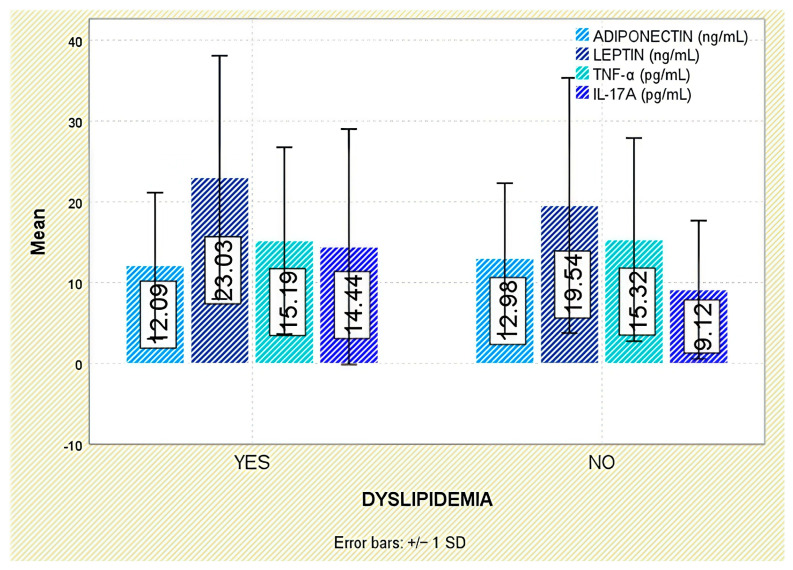
Serum levels of adiponectin, leptin, TNF-α, and IL-17A in patients with and without dyslipidemia. Error bars represent standard deviation.

With respect to comorbidities, leptin concentrations were significantly elevated in patients with arterial hypertension (*p* = 0.003; Figure 10). Although leptin tended to be higher in those with dyslipidemia, the difference was not statistically significant (*p* = 0.140; Figure 11).

#### 3.2.2. Adiponectin in axSpA

In the overall population, mean serum adiponectin levels were 12.66 ± 9.18 ng/mL. Although no statistically significant sex-based differences were observed (*p* = 0.148), women presented with higher mean concentrations (15.24 ± 11.00 ng/mL) compared to men (11.66 ± 8.24 ng/mL).

Adiponectin levels showed no significant variation with HLA-B27 status (*p* = 0.332), nor across body mass index categories (*p* = 0.151; see Supplementary Figure S1).

**Figure 12:**
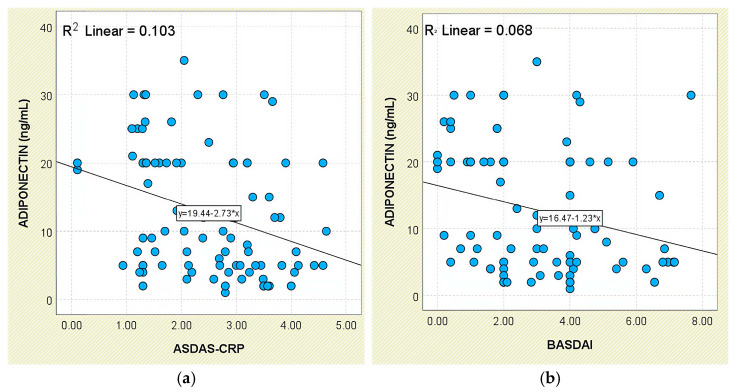
Correlation between serum adiponectin levels and disease activity scores in patients with axSpA. (**a**) Adiponectin vs. ASDAS-CRP; (**b**) adiponectin vs. BASDAI.

In relation to disease activity, serum adiponectin exhibited a significant inverse relationship with ASDAS-CRP scores (r = −0.321, *p* = 0.002; Figure 12a). This pattern was also reflected in group comparisons: mean levels increased from 14.47 ± 9.55 ng/mL in patients with inactive disease to 18.84 ± 8.32 ng/mL in those with low activity, then declined to 9.95 ± 9.00 ng/mL among those with high or very high disease activity (Supplementary Figure S2).

A similar inverse trend was noted in relation to BASDAI scores, with a weak but statistically significant negative correlation (r = −0.261, *p* = 0.013; Figure 12b). Although patients with active disease (BASDAI ≥ 4) had lower mean adiponectin levels (10.76 ± 8.16 ng/mL), group differences did not achieve statistical significance (*p* = 0.145; Supplementary Figure S3).

**Figure 13:**
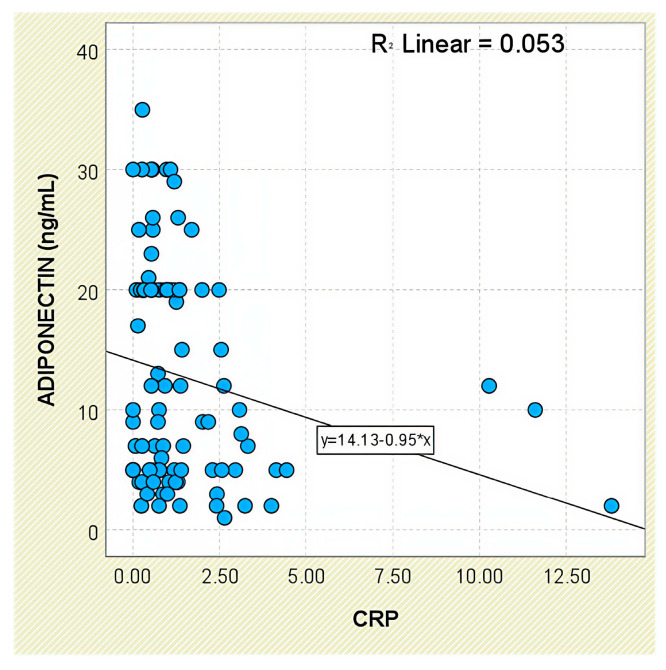
Correlation between serum adiponectin levels and CRP values in patients with axSpA.

Regarding systemic inflammation markers, adiponectin levels were inversely correlated with CRP concentrations (r = −0.230, *p* = 0.030; Figure 13). The correlation with ESR was weaker and did not reach statistical significance (r = −0.191, *p* = 0.073).

Analysis of functional outcomes revealed no significant associations between serum adiponectin and either the Schober test (*p* = 0.160) or fingertip-to-floor distance (*p* = 0.349; Figure 3 and Figure 4).

Radiographic analysis indicated a significant inverse correlation between adiponectin levels and syndesmophyte presence (r = −0.293, *p* = 0.005; Figure 5). In addition, lower adiponectin levels were associated with more severe radiographic damage based on mSASSS (*p* = 0.021; Figure 6).

When examining extra-articular manifestations (psoriasis, IBD, acute anterior uveitis), differences in adiponectin concentrations were observed but did not reach statistical significance (Figure 7, Figure 8 and Figure 9).

Among evaluated comorbidities, patients with arterial hypertension had significantly reduced adiponectin levels (*p* < 0.001; Figure 10). While a trend toward lower values was also noted in dyslipidemic individuals, this did not attain statistical significance (*p* = 0.559; Figure 11).

#### 3.2.3. TNF-Alpha in axSpA

In the study cohort, the mean serum TNF-α concentration was 15.27 ± 12.15 pg/mL. Although values were higher in males (16.03 ± 13.32 pg/mL) than in females (13.32 ± 9.39 pg/mL), this difference did not reach statistical significance (*p* = 0.570). Serum TNF-α levels showed no significant differences by HLA-B27 status (*p* = 0.808) or across BMI categories (*p* = 0.185; Supplementary Figure S1).

**Figure 14:**
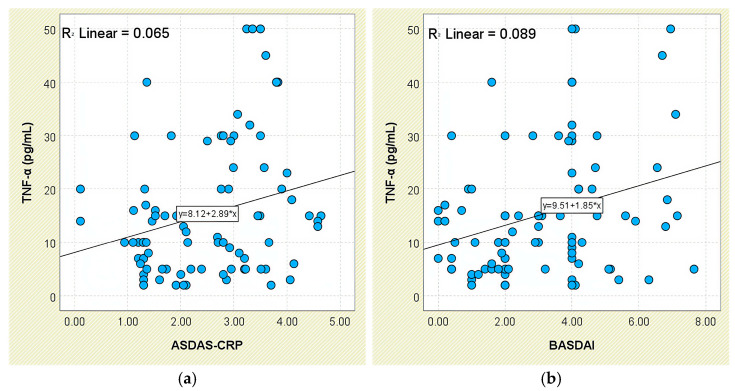
Correlation between serum TNF-α levels and disease activity scores in patients with axSpA. (**a**) TNF-α vs. ASDAS-CRP; (**b**) TNF-α vs. BASDAI.

In relation to disease activity, TNF-α concentrations increased stepwise across ASDAS-CRP categories—from 10.18 ± 6.86 pg/mL in inactive disease to 19.89 ± 14.81 pg/mL in very high disease activity—yet group differences were not statistically significant (*p* = 0.158; Supplementary Figure S2). However, a weak but statistically significant positive correlation was observed between TNF-α and ASDAS-CRP scores (r = 0.256, *p* = 0.016; Figure 14a).

A comparable trend was found for BASDAI: patients with BASDAI ≥ 4 exhibited significantly higher TNF-α levels (19.47 ± 14.74 pg/mL) than those with lower scores (12.14 ± 8.69 pg/mL; *p* = 0.031; Supplementary Figure S3). This was further supported by a moderate positive correlation between TNF-α and BASDAI values (r = 0.298, *p* = 0.005; Figure 14b).

**Figure 15:**
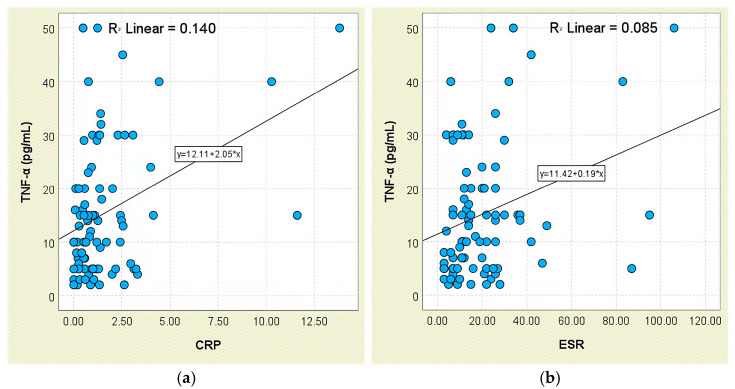
Correlation between serum TNF-α levels and inflammatory biomarkers in patients with axSpA. (**a**) TNF-α vs. CRP; (**b**) TNF-α vs. ESR.

Regarding systemic inflammatory markers, TNF-α levels were higher in patients with elevated CRP (16.76 ± 12.53 pg/mL) and ESR (17.83 ± 14.97 pg/mL), compared to those with normal CRP (11.00 ± 10.02 pg/mL) and ESR (13.61 ± 9.70 pg/mL), though these differences did not reach significance (*p* = 0.053 and *p* = 0.388, respectively). Still, TNF-α showed a weak but significant positive correlation with both CRP (r = 0.374, *p* < 0.001; Figure 15a) and ESR (r = 0.292, *p* = 0.006; Figure 15b).

No significant associations were detected between TNF-α and functional outcomes, including the Schober test (*p* = 0.379) and fingertip-to-floor distance (*p* = 0.993; Figure 3 and Figure 4).

Although TNF-α levels were numerically higher in individuals with moderate or severe syndesmophytes (18.31 ± 14.34 pg/mL) compared to those without (13.36 ± 10.69 pg/mL), the difference was not statistically significant (*p* = 0.288; Figure 5). Likewise, TNF-α levels were not significantly associated with mSASSS scores (*p* = 0.408; Figure 6).

Across extra-articular manifestations—including psoriasis, IBD, and anterior uveitis—TNF-α concentrations did not differ significantly between affected and unaffected individuals (Figure 7, Figure 8 and Figure 9).

Similarly, no significant correlations were found between TNF-α and comorbidities such as arterial hypertension or dyslipidemia (Figure 10 and Figure 11).

However, TNF-α levels were significantly elevated in patients with peripheral involvement, particularly those presenting with enthesitis (*p* = 0.036).

#### 3.2.4. IL-17A in axSpA

The average serum IL-17A concentration in the study cohort was 11.03 ± 11.31 pg/mL. Although female patients exhibited slightly higher levels (12.36 ± 11.51 pg/mL) compared to males (10.52 ± 11.28 pg/mL), the difference was not statistically significant (*p* = 0.409).

No significant variation in IL-17A levels was observed in relation to HLA-B27 status (*p* = 0.100), and concentrations did not differ substantially across BMI categories either (*p* = 0.872; Supplementary Figure S1).

**Figure 16:**
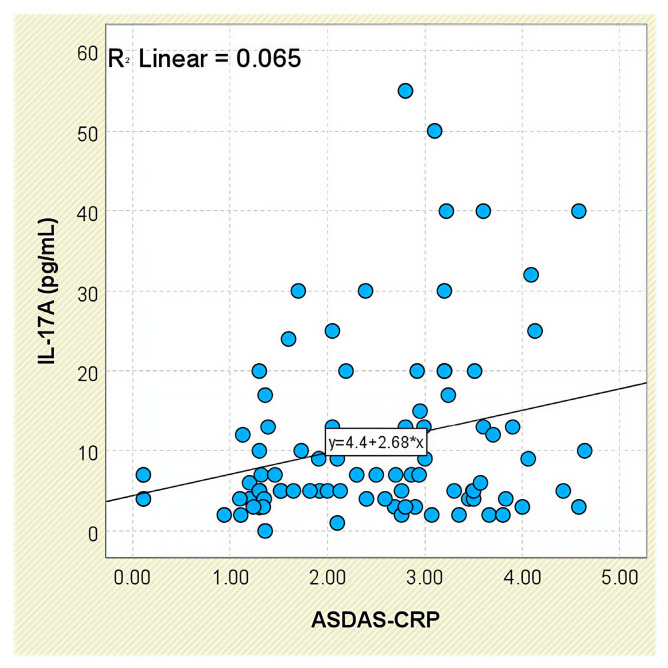
Correlation between serum IL-17A levels and ASDAS-CRP scores in patients with axSpA.

IL-17A concentrations tended to increase with disease activity, as assessed by ASDAS-CRP categories: patients with low activity had a mean of 5.76 ± 4.55 pg/mL, whereas those with very high disease activity reached 13.05 ± 12.51 pg/mL (Supplementary Figure S2). Although intergroup comparisons were not statistically significant (*p* = 0.131), a weak but statistically significant positive correlation was found between IL-17A and ASDAS-CRP scores (r = 0.255, *p* = 0.016; Figure 16).

With respect to BASDAI scores, IL-17A levels showed only minor fluctuations—10.35 ± 11.26 pg/mL in the low-activity group versus 11.95 ± 11.48 pg/mL in the high-activity group—without reaching statistical significance (*p* = 0.602). Similarly, no significant correlation was observed between IL-17A and BASDAI (r = 0.137, *p* = 0.200; Supplementary Figure S3).

Analysis of systemic inflammatory markers revealed no meaningful associations between IL-17A levels and either CRP (*p* = 0.799) or ESR (*p* = 0.330).

Evaluation of functional status did not demonstrate any significant relationship between IL-17A levels and physical mobility parameters, including the Schober test (*p* = 0.295) and fingertip-to-floor distance (*p* = 0.080; Figure 3 and Figure 4).

Radiographic analysis indicated a very weak but statistically significant positive correlation between IL-17A levels and syndesmophyte presence (r = 0.216, *p* = 0.042; Figure 5), as well as with the mSASSS score (r = 0.226, *p* = 0.033; Figure 6).

Interestingly, IL-17A levels were significantly lower in patients with a history of acute anterior uveitis (8.55 ± 15.58 pg/mL) compared to those without this manifestation (11.38 ± 10.67 pg/mL; *p* = 0.018; Figure 9).

No significant associations were identified between IL-17A concentrations and the presence of comorbidities, including arterial hypertension or dyslipidemia (Figure 10 and Figure 11).

## 4. Discussion

This study evaluated the clinical significance of four key biomarkers—leptin, adiponectin, TNF-α, and IL-17A—in patients with axSpA undergoing various pharmacologic treatments. The analysis focused on associations with disease activity, systemic inflammation, radiographic progression, and comorbidities. Our findings highlight distinct but complementary roles of adipokines and proinflammatory cytokines in disease expression, with potential applications for personalized disease monitoring and risk stratification.

### 4.1. Leptin: A Marker of Inflammation and Structural Progression

Leptin consistently reflected inflammatory burden and structural progression, supporting its dual proinflammatory and pro-osteogenic roles, in line with previous findings by Park et al. [18] and Gonzalez-Lopez et al. [19]. Its association with hypertension and psoriasis further suggests overlap between immune and metabolic pathways. Interestingly, elevated leptin-to-BMI ratios in patients with syndesmophytes raise the possibility of a bone-promoting effect independent of adiposity. The higher levels observed in female patients may relate to hormonal or fat-distribution differences, consistent with sex-related disease variability in axSpA. Although leptin was numerically higher in those with dyslipidemia, the lack of statistical significance warrants cautious interpretation.

### 4.2. Adiponectin: An Anti-Inflammatory Modulator

Adiponectin showed inverse associations with inflammatory markers, disease activity, and structural damage, in line with its recognized anti-inflammatory and anti-resorptive functions. These findings align with a meta-analysis by Zhang et al. [20], which reported no significant increase in adiponectin in AS, contrasting with patterns seen in other autoimmune diseases. Its reduced levels in patients with hypertension support a broader cardiometabolic relevance in axSpA. Although a trend toward lower concentrations was observed in dyslipidemic individuals, statistical significance was not reached, possibly due to sample size. Overall, adiponectin may serve as a biomarker for low structural and cardiovascular risk.

### 4.3. TNF-α: A Key Inflammatory Cytokine with Limited Structural Correlation

While TNF-α correlated modestly with disease activity and inflammatory indices, it showed no significant relationship with structural changes—consistent with prior data by Mahendran et al. [21]. Its elevation in patients with enthesitis confirms its role in systemic and peripheral inflammation but also illustrates its limited discriminatory value as a monitoring biomarker for long-term damage. These findings reinforce TNF-α’s role as a therapeutic target rather than a reliable prognostic marker.

### 4.4. IL-17A: Subtle Contributions to Inflammation and Ossification

IL-17A was only modestly associated with disease activity and structural progression, suggesting limited value as a circulating biomarker. Its correlation with syndesmophyte burden may indicate a role in osteoproliferative pathways. Interestingly, we also observed lower IL-17A levels in patients with a history of anterior uveitis, a finding that has not been consistently reported previously. Although this observation should be interpreted cautiously, it may point toward a distinct disease subset and warrants further investigation in larger studies.

### 4.5. Perspectives on Novelty and Clinical Relevance

Although TNF-α and IL-17A are well-characterized therapeutic targets in axSpA, our study adds important nuance by exploring their associations with structural progression and comorbid burden in a real-world cohort. Most notably, our analysis reveals that IL-17A, while only weakly associated with inflammatory markers and activity scores, demonstrated a statistically significant correlation with syndesmophyte formation and mSASSS, suggesting a potential contribution to bone remodeling. Additionally, the observed inverse relationship between IL-17A levels and anterior uveitis represents a novel and unexpected finding that merits further mechanistic exploration, particularly in light of existing therapeutic paradigms targeting IL-17 in axSpA.

More compellingly, leptin and adiponectin, two adipokines with known immunometabolic roles, emerged as more robust correlates of both inflammation and radiographic progression. Notably, the correlation between leptin and structural damage was significant even when adjusted for BMI, suggesting a possible direct pro-osteogenic effect independent of adiposity. The dual behavior of these markers (leptin: proinflammatory and pro-osteogenic; adiponectin: anti-inflammatory and anti-osteogenic) may offer a more integrated understanding of the inflammatory–structural axis in axSpA.

Importantly, both adipokines showed significant associations with comorbid conditions such as arterial hypertension, highlighting their relevance beyond joint inflammation. These findings reinforce the concept that metabolic and inflammatory pathways in axSpA are tightly interconnected. Taken together, our results support the utility of expanding biomarker panels beyond classical cytokines—particularly in patients with cardiometabolic risk profiles or atypical extra-articular manifestations—and advocate for a multidimensional approach to personalized disease monitoring.

### 4.6. Study Limitations

This was a single-center, cross-sectional study, limiting causal inference. The cohort size, though robust, may not capture all disease phenotypes. Additionally, the influence of biologic treatments, particularly anti-TNF agents, on biomarker levels could not be fully adjusted. It is also possible that circulating antigen–antibody complexes interfered with ELISA-based cytokine measurements, particularly for TNF-α and IL-17A, reducing their accuracy. Future longitudinal studies are warranted to validate these associations and assess predictive capacity over time.

### 4.7. Future Directions

Further research should explore the dynamics of these biomarkers in response to treatment, their performance in combination with imaging and genetic markers, and their value in stratifying patients for personalized therapeutic pathways.

## 5. Conclusions

**Figure 17:**
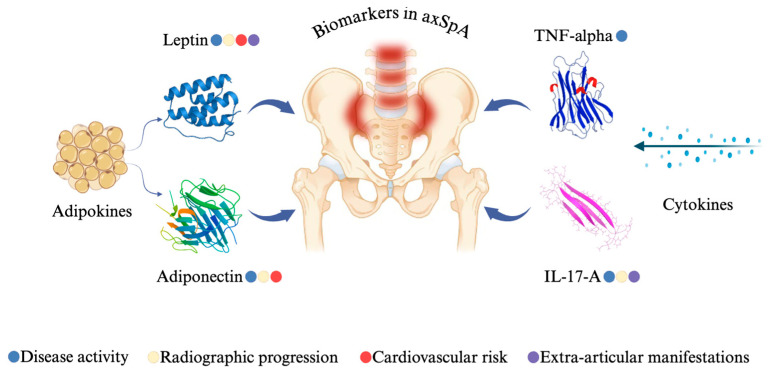
Biomarker significance in axSpA patients from our study.

In conclusion, this study provides comprehensive insights into the role of several key biomarkers—leptin, adiponectin, TNF-alpha, and IL-17A—in the context of axSpA (Figure 17). Our findings demonstrate that serum leptin levels are significantly associated with higher disease activity, inflammatory markers, and radiographic progression, particularly in female patients, suggesting a possible sex-specific contribution of this adipokine to disease pathophysiology. Conversely, adiponectin levels were inversely correlated with disease activity and structural damage, supporting its potential anti-inflammatory and protective role in axSpA. While TNF-alpha did not show significant associations with radiographic progression, it was elevated in patients with peripheral involvement such as enthesitis, reinforcing its relevance in both axial and peripheral disease manifestations. IL-17A levels showed a modest but significant correlation with disease activity and radiographic severity, pointing to a role in inflammation and osteoproliferation. Collectively, these results underline the heterogeneity of biomarker profiles in axSpA and support their potential value in enhancing disease assessment, identifying high-risk patients, and guiding more tailored therapeutic approaches. Further prospective studies are warranted to validate these associations and to explore their applicability in clinical practice.

## Figures and Tables

**Table 1:** Patient demographics, disease-related characteristics, and comorbidities.

	Characteristics	AxSpA (n = 89)
**Demographic data**	Age (years)	50.49 ± 12.48
	Male patients, n (% of 89)	64 (71.9%)
	BMI, median (IQR), kg/m^2^	26.95 ± 5.31
	Smokers (current, ex), n (%)	25 (28.1%)
**Genetic profile**	HLA-B27-positive, n (%)	46 (51.7%)
**Disease-related data**	Disease duration, months, median (IQR)	114.31 ± 103.79
	Peripheral arthritis, n (%)	15 (16.9%)
	ASDAS-CRP, mean ± SD	2.48 ± 1.08
	BASDAI (0–10), mean ± SD	3.11 ± 1.96
	Lumbar flexion (Schober test) (cm)	13.03 ± 1.86
	Finger-to-floor distance (cm)	23.38 ± 15.60
**Inflammatory biomarkers**	CRP (mg/dL)	1.54 ± 2.21
	ESR (mm/h)	20.79 ± 19.13
	TNF-α, pg/mL, median (IQR)	15.27 ± 12.15
	IL-17, pg/mL, median (IQR)	11.03 ± 11.32
**Metabolic biomarkers**	Leptin (ng/mL, median (IQR)	20.80 ± 15.52
	Adiponectin, ng/mL, median (IQR)	12.66 ± 9.18
**Lipid profile parameters**	TC (mg/dL)	200.14 ± 45.1
	LDL-C (mg/dL)	120.69 ± 36.03
	Triglycerides (mg/dL)	122.45 ± 73.95
**Radiographic assessment**	mSASS (0-72), mean ± SD	23.76 ± 28.28
**Extra-articular manifestations and comorbidities**	Patients with syndesmophytes, n (%)	39 (43.82%)
	Uveitis history, n (%)	11 (12.4%)
	IBD history, n (%)	6 (6.7%)
	Psoriasis history, n (%)	5 (5.6%)
	Hypertension history, n (%)	29 (32.6%)
	Dyslipidemia history, n (%)	32 (36%)
	Diabetes mellitus history, n (%)	15 (16.9%)
**Current treatment**	NSAIDs	27 (30.3%)
	csDMARDs	5 (5.6%)
	ANTI-TNF-alpha	38 (42.7%)
	ANTI-IL-17A	11 (12.4%)
	ANTI-JAK	6 (6.7%)

Characteristics are presented as mean ± standard deviation unless indicated otherwise. *ASDAS*, Ankylosing Spondylitis Disease Activity Score; *BASDAI*, Bath Ankylosing Spondylitis Disease Activity Index; *BMI*, body mass index; *CRP*, C-reactive protein; *ESR*, Erythrocyte Sedimentation Rate; *TNF-α*, tumor necrosis factor-alpha; *IL-17*, interleukin 17; *mSASSS*, modified Stoke Ankylosing Spondylitis Spine Score; *TC*, total cholesterol; *LDL-C*, Low-Density Lipoprotein Cholesterol.

## Data Availability

The data presented in this study are available on request from the corresponding author.

## Supplementary Materials

The following supporting information can be downloaded at: https://www.mdpi.com/article/10.3390/jcm14155605/s1, Figure S1: Mean serum levels (±1 SD) of adiponectin, leptin, TNF-α, and IL-17A stratified by BMI category, Figure S2: Mean serum levels (±1 SD) of adiponectin, leptin, TNF-α, and IL-17A stratified by ASDAS-CRP score categories, Figure S3: Mean serum levels (±1 SD) of adiponectin, leptin, TNF-α, and IL-17A stratified by BASDAI score categories.

## Abbreviations

The following abbreviations are used in this manuscript:

SpA	Spondyloarthritis
NSAIDs	Nonsteroidal Anti-Inflammatory Drugs
axSpA	Axial Spondyloarthritis
r-axSpA	Radiographic Axial Spondyloarthritis
AS	Ankylosing Spondylitis
nr-axSpA	Non-radiographic Axial Spondyloarthritis
TNF-α	Tumor Necrosis Factor-alpha
IL	Interleukin
JAK	Janus kinase
ASDAS-CRP	Ankylosing Spondylitis Disease Activity Score based on C-reactive protein
BASDAI	Bath Ankylosing Spondylitis Disease Activity Index
mSASSS	Modified Stoke Ankylosing Spondylitis Spine Score
CRP	C-reactive protein
ESR	Erythrocyte Sedimentation Rate
NF-κB	Nuclear Factor kappa-light-chain-enhancer of activated B cells
MAPKs	Mitogen-Activated Protein Kinases
TMB	Tetramethylbenzidine
