# Supplementary material for: Biomarkers of Inflammation and Radiographic Progression in Axial Spondyloarthritis: A Clinical Evaluation of Leptin, Adiponectin, TNF-α, and IL-17A
Source: J Clin Med. 2025 Aug 7;14(15):5605. doi: 10.3390/jcm14155605 (PMC12347626; doi:10.3390/jcm14155605)
Supplement: Supplementary file 1 [file jcm-14-05605-s001.zip › jcm-3697625-supplementary.pdf]

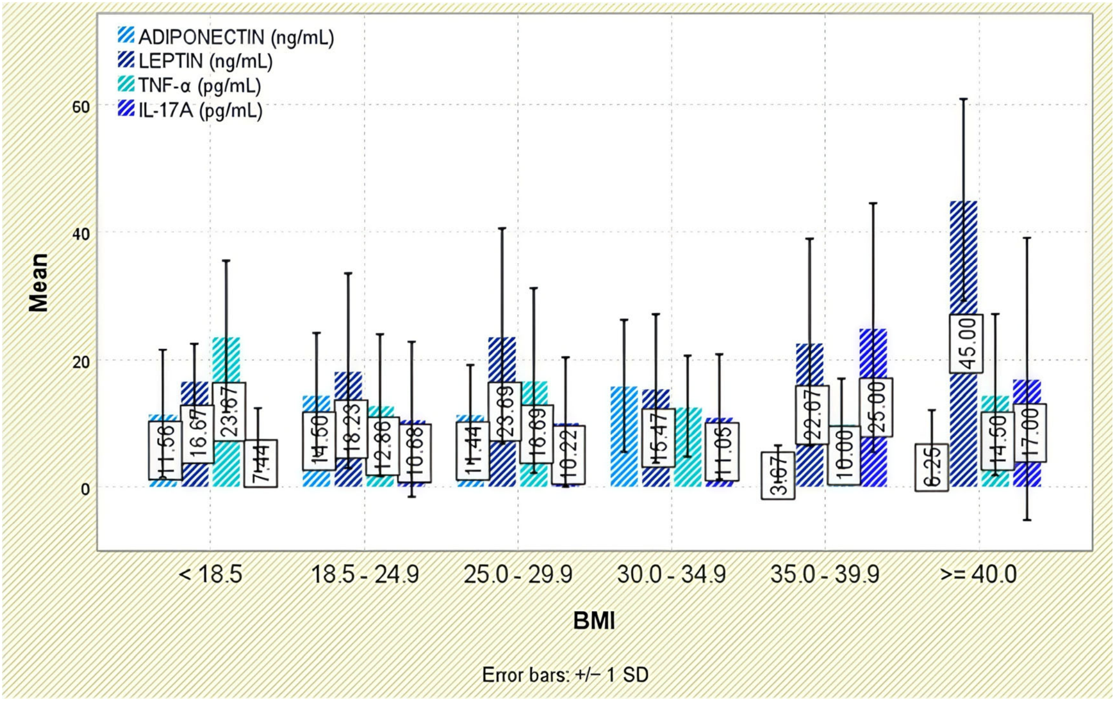

Figure S1. Mean serum levels (±1 SD) of adiponectin, leptin, TNF-α, and IL-17A stratified by BMI category.

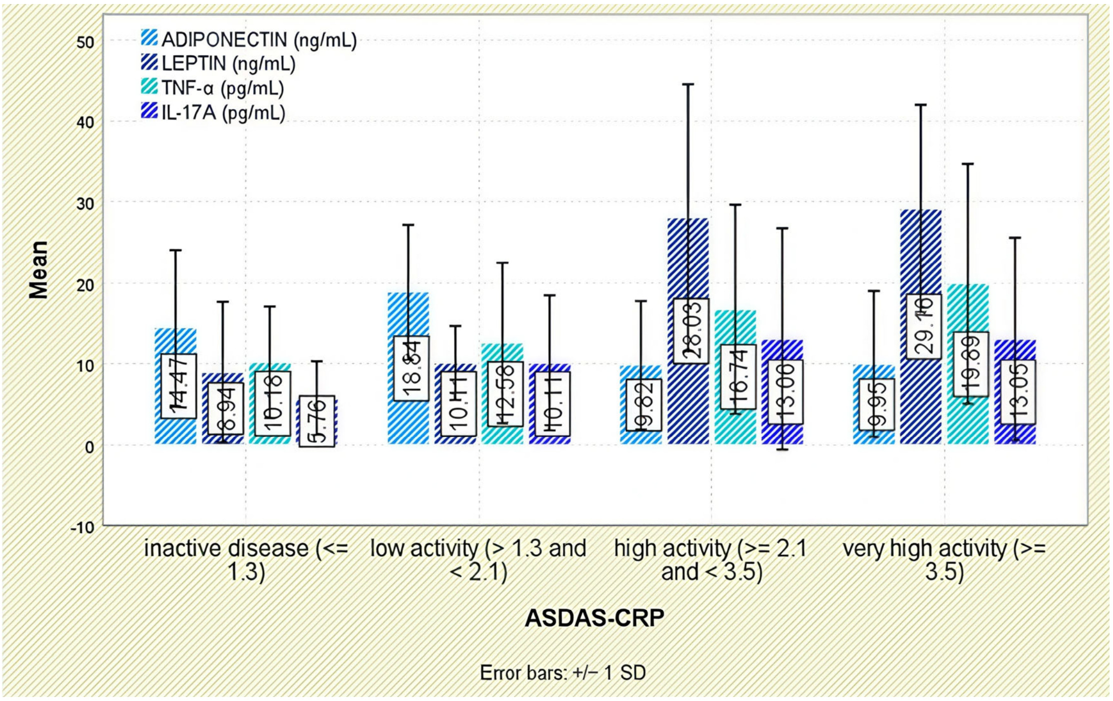

Figure S2. Mean serum levels (±1 SD) of adiponectin, leptin, TNF-α, and IL-17A stratified by ASDAS-CRP score categories.

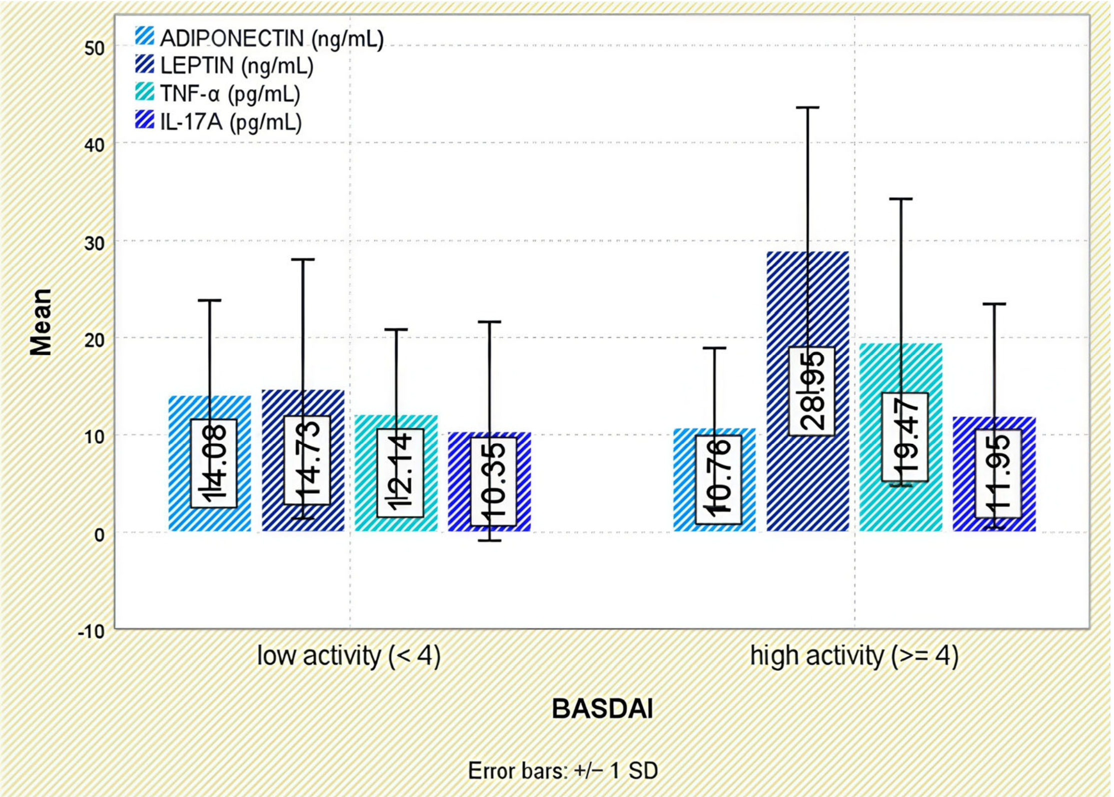

Figure S3. Mean serum levels (±1 SD) of adiponectin, leptin, TNF-α, and IL-17A stratified by BASDAI score categories.
